# High risk, high gain? Trade-offs between growth and resistance to extreme events differ in northern red oak (*Quercus rubra* L.)

**DOI:** 10.3389/fpls.2024.1374498

**Published:** 2024-04-05

**Authors:** Jonathan M. Kormann, Ernst van der Maaten, Mirko Liesebach, Katharina J. Liepe, Marieke van der Maaten-Theunissen

**Affiliations:** ^1^ Chair of Forest Growth and Woody Biomass Production, TU Dresden, Dresden, Germany; ^2^ Thünen Institute of Forest Genetics, Grosshansdorf, Germany

**Keywords:** dendroecology, tree rings, climate-growth relationships, provenance trial, introduced species, frost hardiness, drought hardiness

## Abstract

Information about the resistance and adaptive potential of tree species and provenances is needed to select suitable planting material in times of rapidly changing climate conditions. In this study, we evaluate growth responses to climatic fluctuations and extreme events for 12 provenances of northern red oak (*Quercus rubra* L.) that were tested across three trial sites with distinct environmental conditions in Germany. Six provenances each were sourced from the natural distribution in North America and from introduced stands in Germany. We collected increment cores of 16 trees per provenance and site. Dendroecological methods were used to compare provenance performance and establish climate-growth relationships to identify the main growth limiting factors. To evaluate the provenance response to extreme drought and frost events, three site-specific drought years were selected according to the Standardized Precipitation Evapotranspiration Index (SPEI) and 2010 as a year with an extreme late frost event. Resistance indices for these years were calculated and assessed in relation to overall growth performance. We observed a high variation in growth and in the climate sensitivity between sites depending on the prevailing climatic conditions, as well as a high intra-specific variation. Overall, summer drought and low temperatures in the early growing season appear to constrain the growth of red oak. The resistance of provenances within sites and extreme years showed considerable rank changes and interaction effects. We did not find a trade-off between growth and resistance to late frost, namely, fast growing provenances had a high frost hardiness. Further, there was no evidence for a trade-off between growth and drought hardiness. Still, responses to drought or late frost differ between provenances, pointing to dissimilar adaptive strategies. Provenances from introduced (i.e. German) stands represent suitable seed sources, as they combine a higher growth and frost hardiness compared to their North American counterparts. Drought hardiness was slightly higher in the slow-growing provenances. The results provide a better understanding of the variable adaptive strategies between provenances and help to select suitable planting material for adaptive forest management.

## 1 Introduction

The longevity and sessile nature of trees exposes them to diverse climate conditions during their lifetime. Due to climate change, these conditions nowadays not only include the climate variability typical for a specific region but also long- and short-term changes in climate, such as gradual increases in temperature and altered precipitation regimes that may lead to lower water availability in the growing season (IPCC, 2021). In particular, the frequency and severity of extreme events is increasing, which challenges tree populations to cope with harsh conditions, resulting in reduced productivity, crown defoliation or even mortality (Anderegg et al., 2015; Jump et al., 2017). Trees are forced to rapidly adapt to the new conditions or migrate to more advantageous environments (Aitken et al., 2008).

Therefore, knowledge about the tolerance of tree populations to changing climate regimes is essential. Here, dendroecological studies focusing on climate responses of tree species relevant for forest management (Huang et al., 2010; Montwé et al., 2016) are deemed particularly important. Namely, such studies, which use tree rings to analyze the impact of ecological influences such as drought or late frost events on tree growth (Speer, 2011), provide insights into climate-growth relationships and the resilience of trees to climate extremes at wide spatial and temporal scales (Evans et al., 2018; Kannenberg and Maxwell, 2022). Dendroecological studies may not only make an important contribution towards quantifying climatic tolerances of tree species but also of provenances. Hence, tree-ring based studies in provenance trials, established to compare the performance of multiple provenances of a species under different climatic and environmental conditions (Williams et al., 2002), offer a great potential to explore intra-specific variation patterns and to define promising adapted provenances and thus suitable planting material for forest management (Housset et al., 2018).

Tree-ring studies in Europe so far mostly focused on native tree species, e.g., with studies on Norway spruce (Rocha et al., 2021), Scots pine (Taeger et al., 2013), European larch (George et al., 2017), European beech (Stolz et al., 2021) and native oaks (Sohar et al., 2014). As a possible decline in growth and a loss of vigor of these native tree species due to rapid climate change poses serious problems for the resilience of forest ecosystems (Jump et al., 2017), the focus has more recently widened to alternative species. These alternative tree species include introduced (non-native) species and could make an important contribution towards creating more resilient and climate-adapted forests in the future (Thurm et al., 2018; Brus et al., 2019). Northern red oak (*Quercus rubra* L.), hereafter named red oak, is one of those alternative species, promising due to its high growth potential, versatile use of wood and presumably high climate tolerance (Nagel, 2015; Nicolescu et al., 2020). The species is native to North America, though was introduced to Europe already over 300 years ago. Nowadays, red oak covers 350,000 ha in European forests and is the most widespread introduced deciduous species in Germany (55,000 ha) (Nicolescu et al., 2020; BMEL, 2022). In its natural distribution, red oak covers a broad climatic and altitudinal gradient ranging up to 1,680 m a.s.l (Nagel, 2015; Nicolescu et al., 2020). The precipitation regime varies between 760 and 2,080 mm and the mean annual temperature reaches 4°C in the northern and 16°C in the most southern part (Sander, 1990). In Europe, and particularly in Germany, red oak grows across a large temperature gradient similar to that of its natural distribution, but it often experiences less precipitation (minimum 500 mm) (Nagel, 2015; Nicolescu et al., 2020). Compared to native oaks (*Quercus robur* L. and *Q. petraea* (Matt.) Liebl.), it is suggested to have a higher tolerance to environmental stress and to be less affected by water deficits due to lower water consumption and a higher stomatal density (Dyderski et al., 2020; Nicolescu et al., 2020). Supposedly, red oak is tolerant to winter and spring frosts due to a later bud break, whereas it appears to be highly sensitive to autumn frosts (Nicolescu et al., 2020).

Despite the climatic potential of red oak, only few studies have addressed its climatic response and tolerance to extreme weather events, and thereby found contrasting results. A study conducted in the northern natural distribution, for example, revealed similar climatic responses of ring width and vessel characteristics (Tardif and Conciatori, 2006b) with the strongest effect of drought in the early growing season (Tardif et al., 2006), whereas a study conducted in the introduced range (Latvia) showed that water deficit in late summer was the most influential factor (Matisons et al., 2015). These contrasting findings illustrate that growth responses of red oak to climate fluctuations and extremes, such as drought and late frost, are not well understood. Overall, more knowledge about the adaptive potential of red oak, especially in its introduced range, is needed at the species and provenance level.

To elucidate the climate sensitivity of red oak in Central Europe, we perform dendroecological analyses on three sites of a provenance trial in Germany that are characterized by distinct environmental conditions and contain red oak provenances from the natural distribution as well as from introduced stands in Germany. A previous forest mensuration study at these sites revealed that the German red oak provenances outperformed those from the natural distribution, especially in environments with higher water availability (Kormann et al., 2023). This result poses the question whether a trade-off exists between growth and resistance to extreme drought and/or late frost events with German provenances following a ‘high risk, high gain’-strategy: We hypothesize that the higher growth of introduced provenances is coupled with a lower resistance to extreme conditions as compared to their North American counterparts. To explore this strategy, we sampled increment cores to (1) establish climate-growth relationships and identify differences in climate responses between provenances and sites. Further, we (2) examine the growth response to drought and late frost events using resistance indices. These were compared with the absolute growth performance to answer the question whether trade-offs exist. Based on the knowledge gained about differences in adaptive strategies between provenances, we (3) identify suitable provenances for the future.

## 2 Material and methods

### 2.1 Study design

**Figure 1:**
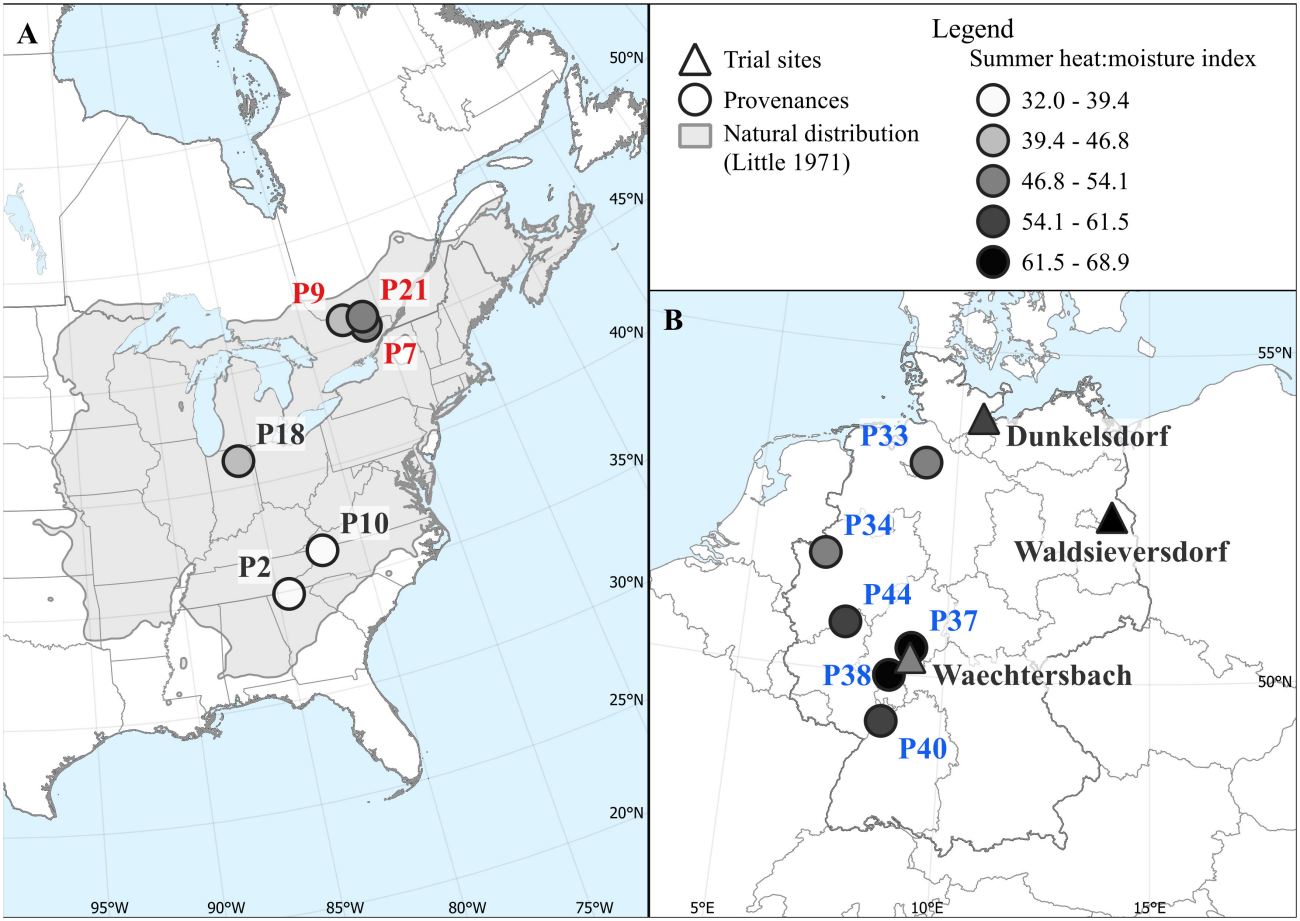
Distribution of provenances (circles) in North America **(A)** and in Germany **(B)** that were planted on the three sites of the provenance trial (triangles). The natural distribution of red oak (Little, 1971) is shown in grey. Provenances and sites are colored according to the summer heat:moisture index (SHM) at their origin to highlight the different moisture conditions during the growing season. Provenances names from the USA, Canada and Germany are colored black, red and blue, respectively; this color scheme is used consistently hereafter when referring to the specific origin of provenances.

We investigated growth responses of red oak using a provenance trial that was established by the Thünen Institute of Forest Genetics in 1991. Both provenances from the natural distribution as well as from introduced stands from Germany were planted on four sites, from which one site was abandoned due to a high mortality caused by rodents. The three remaining sites are located in northern (Dunkelsdorf, DDF), eastern (Waldsieversdorf, WSD) and central (Waechtersbach, WBH) Germany (**Figure 1**). All sites were set up as a randomized complete block design with four replications; for a detailed overview see Liesebach and Schneck (2011). Climatically, the sites can be described as humid (Waechtersbach, Dunkelsdorf) and continental (Waldsieversdorf). Overall, the sites cover a precipitation gradient ranging from 862 mm in Waechtersbach to 586 mm in Waldsieversdorf (**Table 1**). The mean of the maximum temperature of the warmest and coldest month ranges from 21.8°C (Dunkelsdorf) to 23.6°C (Waldsieversdorf) and from -0.6°C to -1.7°C respectively (**Supplementary Figure 1**). The sediment material of the soil varies between silt in Waechtersbach, sandy loam in Dunkelsdorf and sand with clay bands in Waldsieversdorf.

**Table 1:** Overview of sites, mean annual temperature (MAT), mean annual precipitation (MAP), summer heat:moisture index (SHM) and number of measured trees per site (N trees) (WBH, Waechtersbach; DDF, Dunkelsdorf; WSD, Waldsieversdorf).

Site	Latitude	Longitude	Altitude (a.s.l.)	MAT [°C]	MAP [mm]	SHM [C°/µm]	N trees
WBH	50.27° N	09.15° E	330	9.3	862	47	190
DDF	53.97° N	10.60° E	50	9.2	758	53	191
WSD	52.54° N	14.03° E	80	9.7	586	67	192

### 2.2 Tree-ring data

In spring 2022, we collected increment cores of 16 dominant or co-dominant trees per provenance and site (i.e., four individuals per replicate). The 12 provenances sampled (**Figure 1**; **Supplementary Table 1**), originate from introduced stands in Germany as well as from stands in the natural distribution and were selected according to their occurrence on each site (Kormann et al., 2023). In particular, we extracted two 5-mm cores per tree at 1.3 m (diameter at breast height, DBH), resulting in a total number of 1,152 cores. For preparation, the increment cores were air-dried and cut using a WSL microtome (Gärtner and Nievergelt, 2010). High-resolution images were then made using an ATRICS system (Levanič, 2007). Annual ring-width measurements were done on the resulting images and visually and statistically crossdated using the CooRecorder and CDendro software (version 9.6.3, Cybis Elektronik & Data AB, Sweden).

Tree-ring series of individual trees, obtained after averaging the measurements of the two cores per tree, were treated in two different ways. For studying the climate sensitivity of tree growth (cf. Section 2.4), individual series were detrended using a cubic-smoothing spline with a 50% frequency cut-off at 15 years. This procedure removes age and dimension-related trends and accentuates the high-frequent climate signal (Speer, 2011). Individual index series were finally averaged using a bi-weight robust mean to obtain site- and provenance-specific chronologies. Tree-ring series were transformed into basal area increment for visualization of the red oak chronologies, using the *bai.out* function of the *dplR* package and the diameter at breast height (DBH) of each individual tree, thereby reducing dimension-related trends (Bunn, 2008; Bunn, 2010).

In order to assess and validate the quality of our chronologies, chronology statistics were calculated (**Table 2**). The standard deviation indicates the data dispersion around the mean and first-order autocorrelation represents the impact of growth from the previous to the current year (Fritts, 1976). Gleichläufigkeit describes the similarity in growth responses between chronologies from year-to-year (Speer, 2011). The mean inter-series correlation (RBAR) measures the strength of the common signal between series and thus indicates its consistency (Cook and Kairiukstis, 1990). The expressed population signal (EPS) shows how adequate the subsample of series represents the theoretical population (Wigley et al., 1984). For comparability, we calculated chronology statistics for the common overlap period 1999 to 2021, when tree-ring series were available for at least 80% of the trees per site. All tree-ring data processing was done in R using the *dplR* package (Bunn, 2008; Bunn et al., 2023).

**Table 2:** Chronology statistics for the common overlap period from 1999 to 2021 based on detrended provenance chronologies per site: standard deviation (SD), first-order autocorrelation (AR1), Gleichläufigkeit (GLK), series intercorrelation (RBAR) and expressed population signal (EPS).

	P2	P7	P9	P10	P18	P21	P33	P34	P37	P38	P40	P44
Waechtersbach												
SD	0.176	0.169	0.159	0.174	0.187	0.150	0.132	0.142	0.142	0.161	0.141	0.145
AR1	-0.102	-0.212	-0.204	-0.024	-0.114	-0.137	-0.197	-0.144	-0.212	-0.105	-0.095	-0.144
GLK	0.785	0.783	0.753	0.750	0.765	0.754	0.797	0.809	0.769	0.752	0.808	0.763
RBAR	0.622	0.546	0.500	0.475	0.585	0.571	0.592	0.605	0.589	0.543	0.540	0.632
EPS	0.958	0.948	0.933	0.927	0.948	0.952	0.959	0.961	0.956	0.943	0.949	0.957
Dunkelsdorf												
SD	0.149	0.160	0.167	0.177	0.185	0.160	0.133	0.128	0.147	0.131	0.142	0.132
AR1	0.075	0.121	0.121	0.035	0.060	0.087	0.027	0.048	0.108	0.082	0.098	0.057
GLK	0.741	0.771	0.699	0.722	0.647	0.718	0.729	0.740	0.690	0.715	0.714	0.736
RBAR	0.511	0.533	0.433	0.500	0.471	0.429	0.528	0.477	0.395	0.461	0.478	0.529
EPS	0.926	0.945	0.901	0.929	0.899	0.882	0.931	0.916	0.887	0.917	0.917	0.936
Waldsieversdorf												
SD	0.227	0.179	0.168	0.206	0.234	0.181	0.200	0.180	0.178	0.202	0.185	0.188
AR1	-0.352	-0.203	-0.204	-0.225	-0.319	-0.192	-0.306	-0.258	-0.364	-0.333	-0.380	-0.381
GLK	0.843	0.740	0.738	0.787	0.883	0.711	0.818	0.746	0.805	0.767	0.813	0.836
RBAR	0.669	0.495	0.486	0.597	0.682	0.481	0.529	0.502	0.638	0.585	0.639	0.679
EPS	0.963	0.927	0.925	0.947	0.968	0.911	0.931	0.934	0.961	0.948	0.958	0.962

### 2.3 Climate data

Climate data of each site were extracted as monthly means of maximum, mean and minimum daily temperature and monthly precipitation from a 1 × 1 km gridded database provided by the German Weather Service (DWD Climate Data Center, 2022a, 2022b, 2022c, 2022d). The data refers to the period 1991-2021 to cover the timespan of the provenance trial. As an indicator of drought, we calculated the Standardized Precipitation Evapotranspiration Index (SPEI) based on temperature and precipitation data using the R package *SPEI* (Beguería and Vicente-Serrano, 2017). The multiscalar drought index SPEI integrates a climatic water balance and calculates the effect on drought severity for specific time scales (Vicente-Serrano et al., 2010). For the calculation of SPEI, we used a timescale of 6 months to represent the water availability for tree growth.

### 2.4 Data analysis

The climate sensitivity of tree growth was studied by establishing climate-growth relationships as bootstrapped correlation coefficients between the site- and provenance-specific chronologies and monthly climate variables (temperature, precipitation and SPEI). Correlations were calculated over a 16-month window from previous year June to current year September using the R package *treeclim* (Zang and Biondi, 2015). To explore the climate sensitivity at different time scales, bootstrapped correlations were also calculated for seasonal variables.

Besides analyzing long-term relationships between climate and tree growth, we compared growth resistance of red oak to extreme drought and late frost events. Following a classification after McKee et al. (1993), years were considered as drought years in case SPEI values in May, June or July (when most tree growth occurs; cf. van der Maaten et al., 2018) exceeded thresholds of -1.5 (severe) or -2.0 (extreme; for an overview of the driest years, see **Table 3**; **Supplementary Figure 2**). In addition, we selected 2010 as a year with an extreme late frost event in May for all sites (**Supplementary Figure 3**; see also Buras et al., 2020). In contrast to resilience, which describes the ability to recover from an extreme event and reach pre-disturbance growth, we analyzed resistance indices for each extreme year to quantify provenance-specific growth reductions associated with climatic extremes (Lloret et al., 2011). Resistance was calculated as the ratio between growth in the extreme year and in the previous year with detrended data to reduce high age-related trends, using the R package *pointRes* (van der Maaten-Theunissen et al., 2015). To test for significant differences between provenances and sites, we established linear models using resistance to drought and late frost respectively as response. Finally, to test our hypothesis, we averaged the site-specific resistance in the extreme years per provenance and correlated it with the absolute growth performance, expressed through diameter at breast height. Data analysis was performed in the R programming environment (R Core Team, 2022).

**Table 3:** Analyzed extreme years per site.

Site	Drought years				Late frost event	T min May [°C] (mean 1991-2021)
	Severe	max. SPEI (month)	Extreme	max. SPEI (month)		
WBH	2011	-1.66 (May)	2015	-2.09 (July)	2010	6.1 (7.9)
	2014	-1.73 (June)				
DDF	2003	-1.79 (July)	2018	-2.19 (July)	2010	5.7 (7.2)
	2009	-1.80 (May)				
WSD	2003	-1.92 (May)	2016	-2.00 (May)	2010	6.8 (7.9)
			2018	-2.00 (June)		

Drought years are separated into severe (SPEI: -2.0 to -1.5) and extreme (SPEI < -2.0) drought events during the month May to July. 2010 was selected as a year with an extreme late frost event at all sites (WBH, Waechtersbach; DDF, Dunkelsdorf; WSD, Waldsieversdorf).

## 3 Results

### 3.1 Differences in spatial-temporal growth variation

The chronology characteristics displayed distinct differences in spatial-temporal growth variation between provenances and sites (**Table 2**). Standard deviation was highest in Waldsieversdorf followed by Waechtersbach and Dunkelsdorf, which points to variation in the common response of provenances to short-term climate changes. The autocorrelation of the provenance chronologies is generally low with a maximum of 0.12 (P7 and P9) in Dunkelsdorf, which had the highest values of the three sites. In Waechtersbach and Waldsieversdorf, autocorrelation is negative, displaying low carry-over effects from previous years’ growth. In general, Gleichläufigkeit and RBAR values were high, indicating a strong common growth signal of provenances within sites. Waldsieversdorf had the highest RBAR values (i.e. provenances from Germany and United States), showing a strong common response to climatic variation and the most coherent growth pattern. Likewise, all EPS scores exceeded the common applied threshold of 0.85, representing an adequate and common signal of the provenance chronologies.

### 3.2 Climate-growth relationships

**Figure 2:**
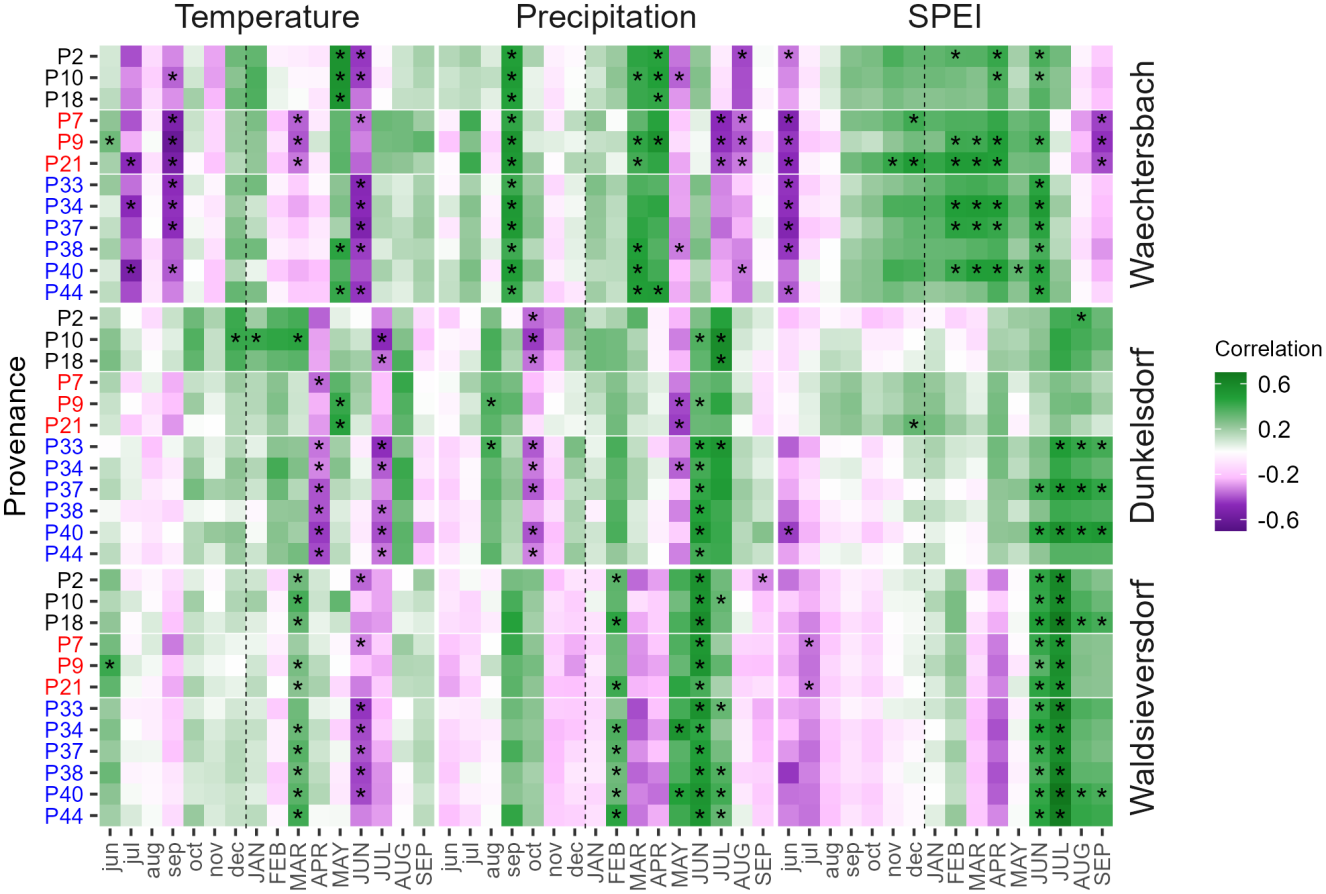
Heatmap with bootstrapped correlation coefficients between provenance chronologies (y-axis) and monthly climate data (temperature, precipitation and SPEI) from previous-year June (jun) to current-year September (SEP). Sites are arranged according to the precipitation gradient from wet (Waechtersbach) to dry (Waldsieversdorf). Correlation coefficients range from negative (violet) to positive (green) values and asterisks indicate significant correlations.

Climate-growth relationships revealed distinct differences between sites and provenances (**Figure 2**). We found a significant positive correlation between growth and SPEI as well as negative correlations with mean and maximum temperature in June/July (**Figure 2**; **Supplementary Figure 4**). Furthermore, June precipitation was positively correlated at all three sites with increasing (significant) dependencies along the precipitation gradient from wet (Waechtersbach) to dry (Waldsieversdorf), identifying summer drought as one of the main climatic factors controlling tree growth of red oak. The highest drought sensitivity was found in Waldsieversdorf with significant positive correlations for SPEI in June and July for all provenances. In Dunkelsdorf, summer drought sensitivity was highest for German provenances, while most provenances from the natural distribution in Waechtersbach showed a strong correlation with spring drought, as indicated by positive correlations with SPEI in April (**Figure 2**; **Supplementary Figure 5**). In Waechtersbach, climate-growth relationships furthermore showed strong dependencies of red oak on moisture conditions in September of the previous year, as indicated by positive correlations with precipitation and negative correlations with temperature (**Figure 2**). In Dunkelsdorf, provenances from Germany and the United States had negative correlations with the moisture conditions in October of the previous year, while the influence of the previous year was not verifiable in Waldsieversdorf. Correlations with early-growing (March to May) mean and maximum temperatures differed considerably between sites. While provenances in Waechtersbach and Waldsieversdorf showed significantly positive correlations in May and March respectively, provenances in Dunkelsdorf had negative correlations in April with significant dependencies of those from Germany (**Figure 2**). Within sites, provenances showed a common response according to their origin (e.g., Canadian provenances in Waechtersbach, German provenances in Dunkelsdorf), whereas the response between sites varied depending on the prevailing conditions.

**Figure 3:**
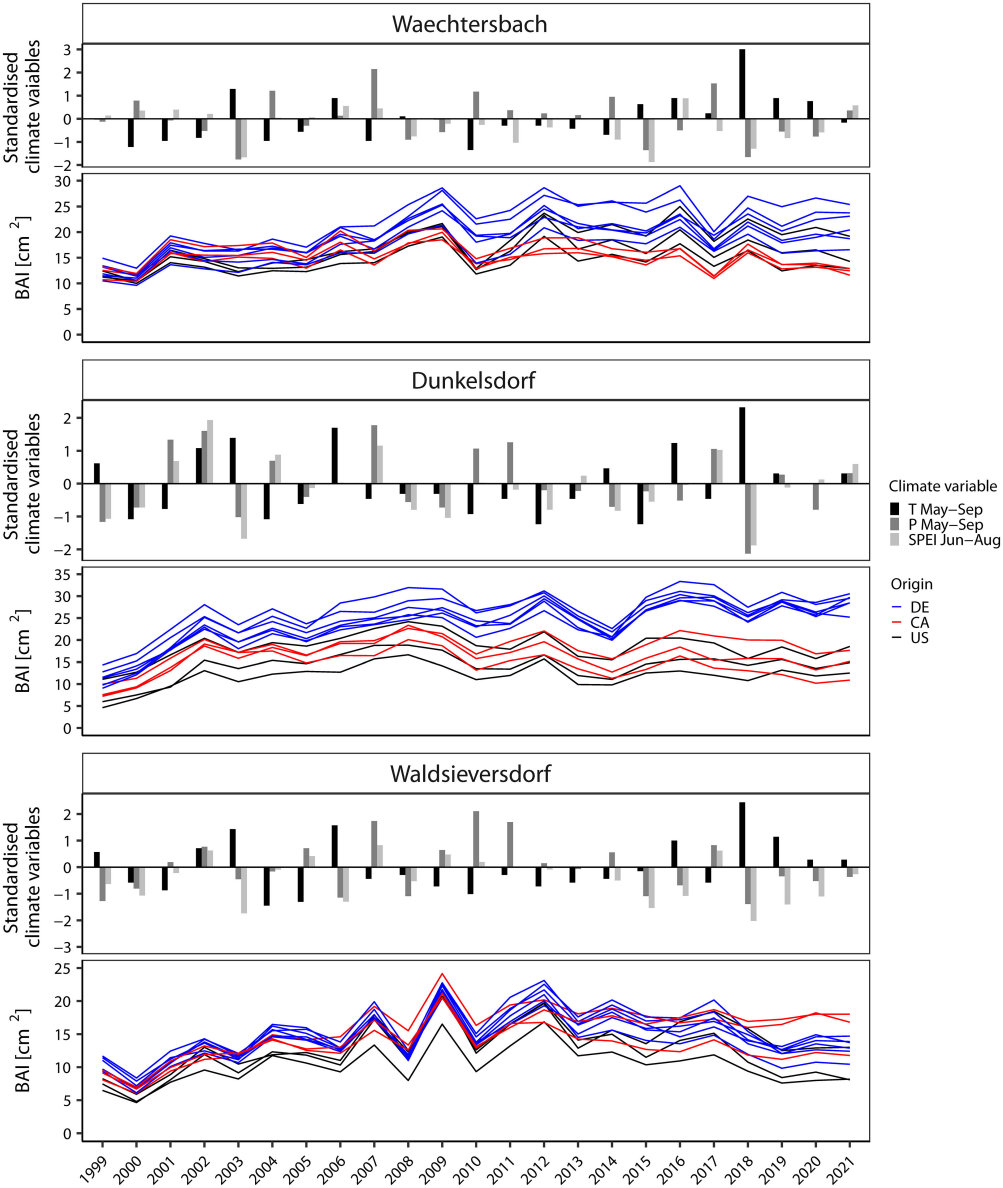
Visual comparison between standardized climate variables and the basal area increment (BAI) [cm²] respectively (note different scales). As climate variables, the precipitation (P) and temperature (T) from May to September as well as SPEI in summer (Jun-Aug) indicate drought and temperature deviations during the growing season. Sites are ordered along the precipitation gradient from wet to dry.

To compare the general growth performance of the provenances as well as to identify years with (and potential climatic cause of) extreme growth responses, we visually compared BAI time series with climate deviations (**Figure 3**). For the latter, we focused on temperature and precipitation from May to September as well as on SPEI in the summer months (June to August). Climate deviations differed between sites according to the specific climate conditions experienced revealing years with a high drought impact (e.g., 2003 and 2018) or low temperatures (e.g., 2010). The BAI series showed a comparatively constant growth pattern over the common overlap period, but the annual increment differed between sites. The highest BAI was found for provenances growing in Dunkelsdorf with a clear differentiation between German provenances and those from the natural distribution, followed by provenances in Waechtersbach, where five of the six German provenances showed the highest growth. In Waldsieversdorf, provenances had visually the most sensitive growth pattern and the overall lowest BAI, especially in the early years. Two provenances from Canada (P7 and P21) stand out with a comparable increment to the German provenances, but with a less sensitive growth pattern. The provenances from the United States had a low BAI on all three sites. Years with growth depressions varied between sites and provenances in frequency and intensity. Only in 2010, provenances on all three sites showed a negative response associated with low temperatures in the growing season. Site-specific drought years with a negative deviation in SPEI and precipitation (2003, 2015, 2018) displayed different responses in BAI series across provenances and sites.

### 3.3 Resistance and tree growth

**Figure 4:**
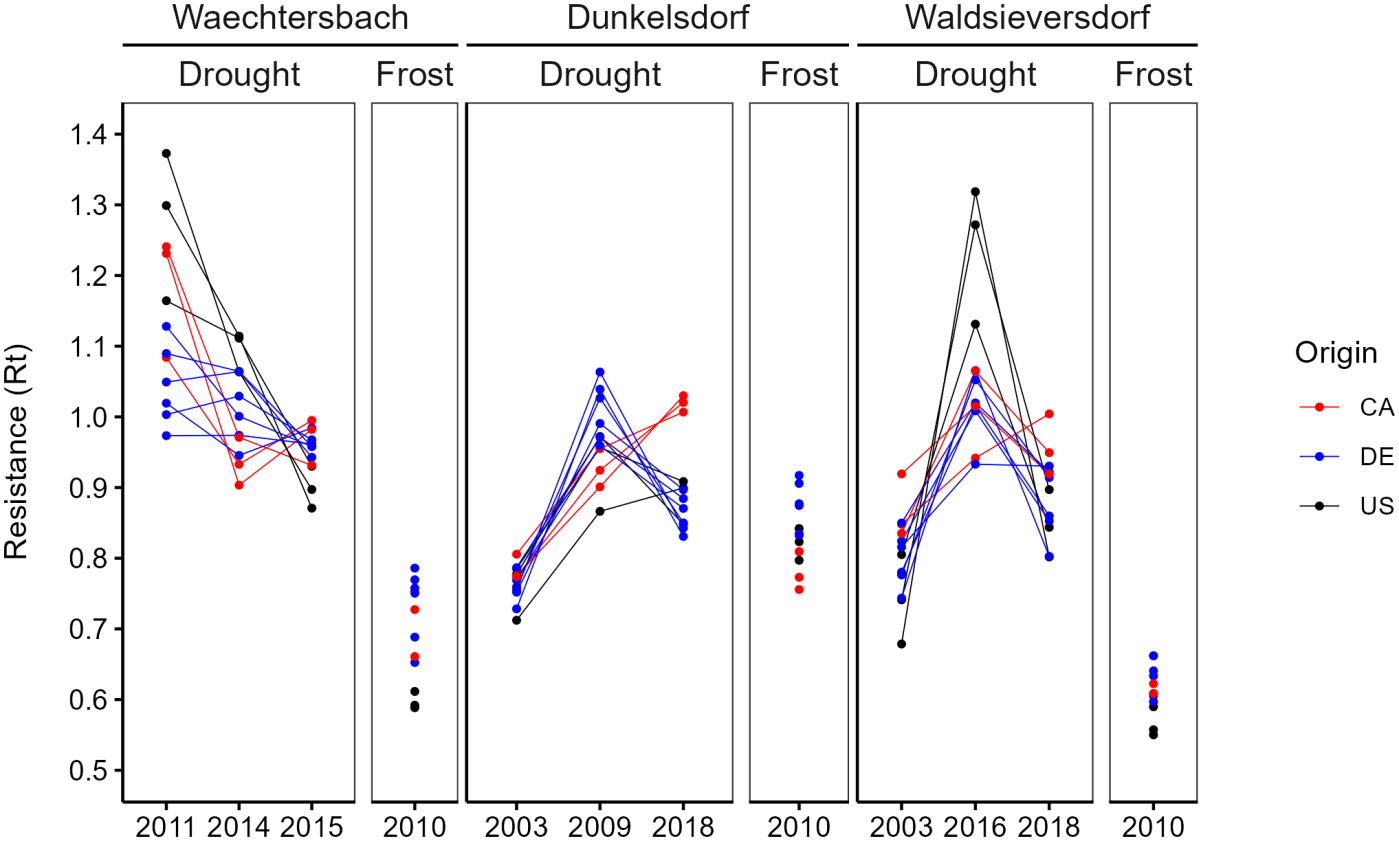
Plasticity of the growth response in site-specific EYs separated between drought and frost events. Lines between EYs are drawn for visibility of interaction and indicate no time-series. Standard errors were omitted for a better visibility (presented in **Supplementary Table 2**).

The visualization of resistance revealed a plasticity of growth responses to drought with a pronounced variation between sites, provenances and drought years (**Figure 4**). Within-year variation was high, with changes in provenance ranks between years. The overall highest resistance to drought was found in Waechtersbach for the severe drought years 2011 and 2014 showing resistance values > 1.0 (**Supplementary Table 2**), which indicates that there was no growth depression during this extreme drought. In the extreme drought year 2015, a growth depression was observed, but with lower variation in resistance between provenances. In contrast, resistance to late frost was lower and German provenances had the highest resistance. In Dunkelsdorf, the lowest resistance was found in the severe drought year 2003 with a low intra-specific variation. The response of the Canadian provenances in 2018 differed from that of their counterparts, with resistance values > 1.0. Resistance in 2010 was lower for the provenances from the natural distribution, while provenances from Germany had a higher frost hardiness. The variation between drought years was most pronounced in Waldsieversdorf. Provenances from the United States showed considerable rank changes between the drought years with no growth decrease in the extreme drought year 2016 (**Supplementary Figure 6**). Across drought years, resistance was lowest in the ‘severe’ drought year 2003. At this site, provenances from Germany had overall the lowest resistance to drought. Provenances in Waldsieversdorf, however, suffered most during the late frost event in 2010 resulting in the lowest resistance overall (**Figure 4**; **Supplementary Table 2**).

The distinct rank changes of the provenances resulted in strong visible interactions between drought years. Overall, provenances from the same geographic origin revealed a common response to drought events, especially in Dunkelsdorf. The growth response during the late frost event resulted in a strong growth depression, with provenances from Germany showing the highest frost hardiness.

The ANOVA revealed significant differences for resistance to late frost between provenances and sites as well as a significant provenance × site interaction with the highest explained variance for sites (**Supplementary Table 3**). For the mean resistance in drought years, provenances and sites showed significant differences with lower explained variance, but no interaction.

**Figure 5:**
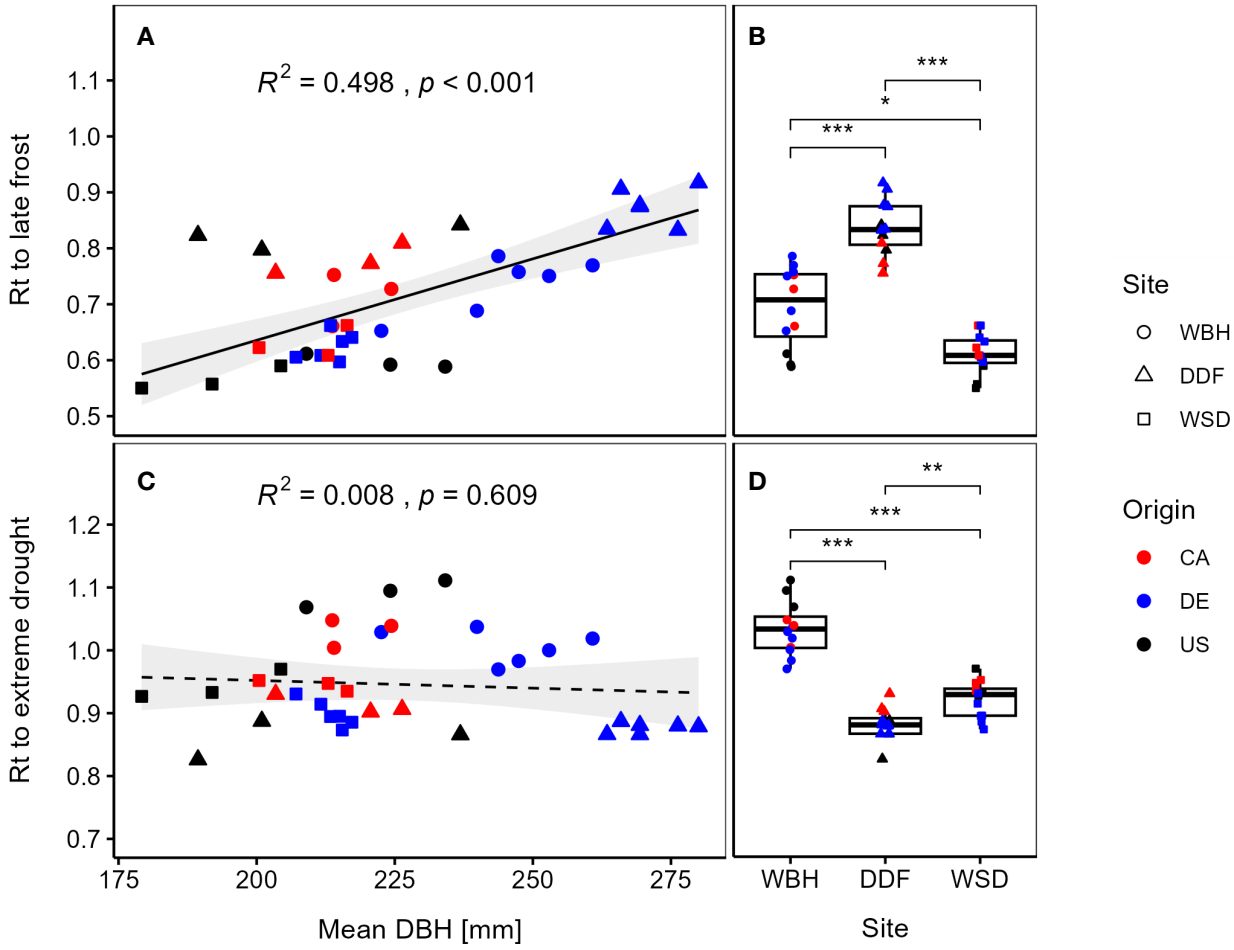
Resistance (Rt) per provenance to late frost (2010) is plotted against the mean diameter at breast height (DBH) **(A)** and at each site **(B)**. In the lower panels, mean Rt for site-specific drought years is plotted against the DBH **(C)** and at each site **(D)**. Point shapes refer to the three sites and line types illustrate significant (solid) or non-significant (dashed) trends. Standard errors of the means were omitted for a better visibility (presented in **Supplementary Table 2**). Significant differences between the site means by Kruskal-Wallis test are indicated by *p < 0.05, **p < 0.01, ***p < 0.001.

We found a significantly positive correlation (R² = 0.498, p < 0.001) between resistance to late frost and diameter at breast height (**Figure 5A**), i.e., provenances with a high absolute growth show a high frost hardiness. The growth differentiation between German and North American provenances was most pronounced in Waechtersbach and Dunkelsdorf, which also showed high resistance values (**Supplementary Figure 6**). Differences between sites were significant with the highest resistance to late frost at the oceanic site Dunkelsdorf, followed by Waechtersbach and Waldsieversdorf, the latter with a lower variation between provenances (**Figure 5B**). On this site, provenances showed similar resistance values with lowest values for provenances from the United States.

However, we found no evidence for a trade-off between growth and mean resistance to drought events (R² = 0.008, p = 0.609) (**Figure 5C**). Although, we observed a decreasing trend between growth and resistance, this trend was not significant and the response of provenances varied across sites with significant differences between all sites. Provenances in Dunkelsdorf showed the strongest growth depression, while the highest resistance values could be observed in Waechtersbach (**Figure 5D**). Overall, the variation of resistance was smaller compared to the resistance to late frost. Similar trends for the response to frost and drought events can be observed when analyzing the resistance indices at the level of individual trees (**Supplementary Figure 7**).

## 4 Discussion

### 4.1 Spatial variation in growth response

We found considerable differences in the growth of provenances between the three sites (**Figures 3**, **5**), suggesting the dependence of environmental effects at spatial scales (Tainter et al., 1990). The different environmental conditions at the sites resulted in differences in absolute growth and/or differences in the climate sensitivity of tree growth. For example, temperature had a greater effect on growth than precipitation in Waechtersbach (i.e. the site with the highest precipitation sum), whereas growth in Dunkelsdorf and Waldsieversdorf showed a strong dependence on moisture conditions and were constrained by summer drought (**Figure 2**). The relationships between growth and environment revealed a decreasing growth trend towards the continental site with more extreme climatic effects and growth rates below average (**Figures 3**, **5**). Compared to Waechtersbach and Waldsieversdorf, we observed higher carry-over effects of nutrient reserves for growth in Dunkelsdorf, likely due to the higher water storage capacity of the soil as indicated by the higher autocorrelation (Speer, 2011). Similar results of site-specific environmental influences affecting tree growth of red oak were obtained in other studies (White et al., 2011; Gillner et al., 2013; Matisons et al., 2015).

The remarkable differences in absolute and secondary growth between introduced provenances and those from the natural distribution as well as a high intra-specific variation are comparable to the previous forest mensuration study by Kormann et al. (2023). The highest growth rates were observed at the oceanic site as well as a growth superiority of German provenances under moisture conditions. Additionally, Canadian provenances had above average growth on the continental site, which was comparable to the growth of German provenances (see **Figure 5C**). Higher growth rates under harsh climatic conditions of provenances originating from the northern part of the natural distribution have previously been demonstrated for red oak (Lindback et al., 2023). Under the continental climate with more extreme temperatures and dry summers, provenances from the United States had growth rates below average. The overall high common response of provenances to climatic effects, indicated by the high Gleichläufigkeit, RBAR and EPS values > 0.85, is likely related to climatic forcing of tree growth (Wigley et al., 1984). However, the plasticity in the growth response to annual climatic fluctuations, especially in drought years, differed between provenances. While provenances from the natural distribution with low growth also showed a low interannual plasticity in their growth response (complacent growth), provenances from Germany with high growth displayed a more sensitive interannual growth pattern (**Figure 3**), indicating different adaptive strategies between the introduced provenances and those from the natural distribution. Comparable results were observed for Douglas fir (Montwé et al., 2015).

### 4.2 Drought and frost events constrain growth

Similar to the spatial effect on tree growth, the analysis of climate-growth relationships revealed differences in the interannual variability of climatic effects. We found summer drought to be a growth-limiting factor at all sites, as indicated by the positive correlations with SPEI in the summer months and the precipitation in June, as well as negative correlations with temperature in June (Waechtersbach and Waldsieversdorf) and July (Dunkelsdorf) (**Figure 2**). The highest drought sensitivity was observed at the continental site with the lowest precipitation and the highest temperatures, which has been demonstrated in other studies with different tree species (Stolz et al., 2021; Weigel et al., 2022). The negative effect of summer drought on tree growth can be explained by a limited access to water during the period of highest cambial activity (van der Maaten et al., 2018). Due to water stress and reduced water uptake, the risk of xylem dysfunction and cavitation increases (Hacke and Sperry, 2001; Fonti et al., 2010), followed by stomatal regulation for water safety and thus reduced photosynthesis and growth rate (Tainter et al., 1990; Sperry, 2003). The correlations with summer drought found in this analysis are comparable to other studies with red oak (Tainter et al., 1990; Tardif and Conciatori, 2006a; Gillner et al., 2013; Matisons et al., 2015) and native oak species (Friedrichs et al., 2009). In addition to summer drought, the negative correlation with precipitation in July and August in Waechtersbach and during the growing season (**Supplementary Figure 5**) suggests that water saturation due to high precipitation during the year further limits growth. At this site, the influence in September of the previous-year had a significant effect on the current year’s growth, suggesting the importance of nutrient reserves and sugar translocation in the main stem, which occurs mainly in red oak during this period (Xu and Griffin, 2006; Gillner et al., 2013). This is also reflected in the significant positive correlation with precipitation and SPEI in previous-year autumn for most of the provenances in Dunkelsdorf.

The identified drought years according to SPEI varied between the three sites. While we detected two consistent years (2003 and 2018; see also Muthers et al., 2017) with severe and extreme effects in Dunkelsdorf and Waldsieversdorf, respectively, the selected drought years in Waechtersbach were completely different (**Table 3**). The response to severe and extreme drought varied between sites and provenances, as indicated by the resistance indices (**Figure 4**). In total, provenances from the natural distribution showed visually a higher mean resistance to drought in Waechtersbach and Waldsieversdorf compared to those from introduced stands. However, there were only minor significant differences between provenances, and the variation in resistance between sites was not very high (**Figure 5D**). Thus, provenances from Canada and the United States showed low growth, but were also less affected by drought and moisture conditions in summer. The reasons for this may be differences in vessel size between fast- and slow-growing provenances, as the variation in vessel diameter affects specific water conductivity and drought-induced cavitation with wide vessels being more susceptible than narrow vessels (Hacke et al., 2017). However, this trade-off is not consistent across sites and the relationship between drought hardiness and absolute growth (DBH) was not significant (**Figure 5C**).

The high variation in the response of the provenances to drought events between drought years may be impacted by an intra-specific growth response (**Figure 4**). The strong interaction effects between drought years can be explained by their difference in intensity and occurrence (different months), and visualized the heterogenous impact of different drought events on tree growth (George et al., 2017). In some drought years (e.g., 2011 in Waechtersbach, 2009 in Dunkelsdorf, 2016 in Waldsieversdorf), we observed a weak to absent interannual response in the BAI series (**Figure 3**). The short-term reaction in growth resulted in negligible growth declines in the following years and commonly observed legacy effect after drought events (Anderegg et al., 2015; Kannenberg et al., 2020) are not clearly identifiable. The weak response to those described drought years in our study has also been observed for native oaks in Europe (Buras et al., 2020). A possible explanation might be the young physiological age of the analyzed trees combined with a smaller canopy compared to mature trees, which may result in a rapid recovery. Therefore, mature trees may suffer more from drought events (Tainter et al., 1990; Buras et al., 2020). However, different adaptive strategies for drought response have been found in red oak between seedlings, juveniles and mature trees (Cavender-Bares and Bazzaz, 2000), with mature trees being less affected by drought than seedlings and juveniles due to a higher photosynthetic capacity and an increased water use efficiency. These findings suggest that our results cannot be extrapolated to other age classes of red oak, as the growth response to climate sensitivity is age dependent and temporally variable (Csilléry et al., 2020; Peltier and Ogle, 2020). As the provenance trial analyzed here consists of relatively young trees, possible changes in the climatic response to extreme events up to the mature period may occur, which may also affect the selection of suitable provenances for planting. Therefore, long-term monitoring of the growth performance of trees in provenance trials is elementary.

In contrast to drought years, which differed between sites, we analyzed the growth response in 2010 for all three sites. This year was characterized by generally low temperatures and a late frost event in May (**Supplementary Figure 3**; see also Buras et al., 2020). During the active growth of trees in this time, frost injuries cause the highest damage with a reduced radial growth caused by freezing-induced embolisms, which lead to a loss in photosynthesis (Howe et al., 2003; Cavender-Bares et al., 2005). As well as in drought years, resistance in 2010 varied significantly between sites with a higher intra-specific variation in Waechtersbach and Dunkelsdorf than in Waldsieversdorf (**Figures 4**, **5B**). We found the highest resistance to late frost in Dunkelsdorf, followed by Waechtersbach and Waldsieversdorf. Overall, introduced provenances showed higher frost hardiness especially in Waechtersbach and Dunkelsdorf, while provenances from the natural distribution had lower resistance to late frost. A previous study by Liesebach and Schneck (2011), on the other hand, showed that in the initial stage of the provenance trial, provenances from Canada had the earliest bud break coupled with the highest frost damage (age 4), whereas provenances from the United States had the latest bud break and were less affected by frost. The differing responses of provenances may emphasize different provenance-specific strategies, e.g. an earlier time of bud break, resulting in a higher risk of frost injuries during a late frost event (Howe et al., 2003). In addition, possible differences between provenances in the timing of wood formation may partly contribute to differences in tree-ring width and the response to frost events. Wood anatomical studies, and xylogenetic analysis in particular, therefore offer promising prospects for future research. Furthermore, we found different responses of provenances between sites to spring temperatures (**Figure 2**), which control the time of flushing and therefore the length of the growing season. This is indicated by the positive response to temperature in March (Waldsieversdorf) and May (Waechtersbach), resulting in an earlier bud break under warmer temperatures. At these sites, we found different dependencies in the response between provenances from Canada and the United States (indicated by different correlations), which may be explained by the earlier flushing of northern provenances in their natural distribution (Sander, 1990; Lindback et al., 2023). Differences in response to spring temperature between introduced provenances and their North American counterparts were predominant in Dunkelsdorf. All introduced provenances showed a significantly negative correlation with temperature in April, which was also found in a previous study with red oak (Gillner et al., 2013). This may be due to the reduced risk of frost injuries caused by late frost events, because of a delayed bud break under cold temperatures, whereas warm temperatures favor bud break (Gunderson et al., 2012). Overall, the higher resistance to late frost point to a possible adaptation in introduced stands. First proof of a possible adaptation to early and late frosts of introduced red oak has already been described by Daubree and Kremer (1993). The origin of the analyzed introduced provenances is unknown in the natural distribution. It is therefore possible that they originate from more predisposed stands. Since, the analyzed period contained only one extreme late frost event, our findings need further examinations to secure the different provenance-specific adaptive strategies.

### 4.3 High risk, high gain?

Surprisingly, our results indicate no trade-off between growth and resistance to frost hardiness, which contradicts the expected ‘high risk, high gain’ -strategy. We found that provenances with a high growth also have a high frost hardiness and in accordance therewith, provenances on the continental site with the lowest productivity had the highest growth depression during the late frost event (**Figure 5A**). Further, we found no (significant) evidence for a trade-off between growth and resistance to drought (**Figure 5C**). However, the drought response varied less between provenances within sites compared to the response to late frost. The absence of a drought hardiness trade-off was also observed for Douglas fir in its natural distribution (Darychuk et al., 2012).

Trade-offs between growth and resistance to drought and frost are a common observation for other tree species, which mainly showed low growth with increasing drought tolerance or frost hardiness (Darychuk et al., 2012; Bennett et al., 2015; Montwé et al., 2016; Csilléry et al., 2020). In this context, a trade-off implies a negative association, resulting in slow growth but high tolerance to environmental extremes, while fast-growing provenances have higher growth depressions under unfavorable conditions (Weih, 2003; Bennett et al., 2015). Reasons for this include exposed canopies and thus a higher transpiration capacity, as well as a greater susceptibility of larger trees to hydraulic stress (Bennett et al., 2015; Grote et al., 2016). Additionally, fast-growing provenances have a higher water use efficiency, which has been found to increase in taller trees (Marshall et al., 2001; Csilléry et al., 2020). The higher water use efficiency may contribute to a higher stress during drought events. This observation implies different strategies between provenances to different climatic impacts (drought and frost). The non-existing trade-off of frost hardiness may be due to a moderate climate at the source location (Guy and Holowachuk, 2001). However, the mean annual temperature and mean annual precipitation varied considerably between provenances from the natural distribution (**Supplementary Table 1**), showing that Canadian provenances experienced harsher climatic conditions with lower temperature and precipitation, which may explain the higher frost hardiness (Kreyling et al., 2015). Since the origin of the introduced stands within the natural distribution is unknown, analyses including the source climate of introduced provenances could result in false interpretations.

## 5 Conclusion

For forest management and plant breeders, provenances with high growth and a high resistance to drought and frost events would be beneficial and have important implications, as they provide suitable reproductive material for future climate conditions (Weih, 2003). The identified provenances with high growth coupled with a higher climatic tolerance underline the recommendation of introduced provenances by Kormann et al. (2023). Under more extreme continental conditions, Canadian provenances may also play a promising role with regard to increased climatic extreme events. However, these results cannot be extrapolated to other environmental conditions, because other local biotic and abiotic factors might have negative implications resulting in maladaptation. Additionally, maladaptation can be more important under future conditions, when frequency and severity of drought and frost events increase (Breda et al., 2006). The non-existing trade-off between growth and frost hardiness points to a possible adaptation of introduced provenances, as also shown by differences in the climate sensitivity to spring temperatures. Because of rising temperatures, bud break is expected to occur earlier in the future and late frost events could become a serious problem for maladapted tree species. Therefore, introduced provenances seem to have an advantage against those from the natural distribution, resulting in lower growth depressions.

However, further investigations like wood anatomical analyses can be helpful in providing information about the cell structure and water use efficiency, and thus explanations of the different responses of provenances and their adaptive strategies. Selecting introduced provenances from the analyzed sites may reduce the risk of frost injuries, which would not jeopardize the drought tolerance in consideration of their superior growth performance. Understanding how populations of introduced tree species adapt to the local environments and respond to increasing drought and frost events will be essential under future climate conditions.

## Data availability statement

The raw data supporting the conclusions of this article will be made available by the authors, without undue reservation.

## Author contributions

JMK: Formal Analysis, Investigation, Methodology, Writing – original draft, Data curation, Validation, Visualization. EM: Methodology, Supervision, Validation, Writing – review & editing. ML: Conceptualization, Funding acquisition, Project administration, Supervision, Writing – review & editing. KJL: Conceptualization, Project administration, Visualization, Writing – review & editing. MMT: Methodology, Supervision, Validation, Visualization, Writing – review & editing.
