# Supplementary material for: High risk, high gain? Trade-offs between growth and resistance to extreme events differ in northern red oak (Quercus rubra L.)
Source: Front Plant Sci. 2024 Apr 5;15:1374498. doi: 10.3389/fpls.2024.1374498 (PMC11026572; doi:10.3389/fpls.2024.1374498)
Supplement: Supplementary file 1 [file DataSheet_1.docx]

Supplementary Material

# Supplementary Tables

Supplementary Table 1: Overview of provenances with coordinates, mean annual temperature (MAT), mean annual precipitation (MAP) and summer heat:moisture index (SHM) at their origin.

| Provenance | Name | Country* | Latitude | Longitude | Altitude (a.s.l.) | MAT [°C] | MAP [mm] | SHM  [°C/μm] |
| --- | --- | --- | --- | --- | --- | --- | --- | --- |
| P2 | Chaahoochee (Georgia) | US | 34.87° N | 84.42° W | 850 | 14.1 | 1849 | 32 |
| P10 | Cherokee (Tennessee) | US | 36.45° N | 82.17° W | 730 | 12.0 | 1311 | 37 |
| P18 | Anderson (Indiana) | US | 41.17° N | 85.67° W | 260 | 10.7 | 1030 | 46 |
| P7 | Constance Bay (Ontario) | CA | 45.50° N | 76.08° W | 260 | 6.2 | 875 | 53 |
| P9 | Atomic Energy (Ontario) | CA | 46.05° N | 77.37° W | 180 | 4.4 | 934 | 43 |
| P21 | Plaines de Kazabazua (Ontario) | CA | 45.93° N | 76.10° W | 210 | 6.0 | 897 | 51 |
| P33 | Bremervoerde (Lower Saxony) | DE | 53.25° N | 09.18° E | 30 | 9.6 | 736 | 50 |
| P34 | Borken (North Rhine-Westphalia) | DE | 51.75° N | 06.83° E | 40 | 11.0 | 799 | 53 |
| P37 | Nidda (Hesse) | DE | 50.42° N | 09.17° E | 240 | 10.2 | 656 | 62 |
| P38 | Moerfelden (Hesse) | DE | 49.98° N | 08.68° E | 85 | 11.2 | 600 | 69 |
| P40 | Wiesloch (Baden-Württemberg) | DE | 49.27° N | 08.58° E | 190 | 10.9 | 674 | 61 |
| P44 | Bornheim (North Rhine-Westphalia) | DE | 50.73° N | 07.50° E | 60 | 11.1 | 747 | 57 |

* CA = Canada, DE = Germany, US = United States of America

Supplementary Table 2: Resistance indices (Rt) in site-specific extreme years as well as mean DBH for each provenance and site. Rt is reported for three drought and one frost year, mean Rt gives the average across drought years. Standard errors are given (± SE).

|  |  | P2 | P10 | P18 | P7 | P9 | P21 | P33 | P34 | P37 | P38 | P40 | P44 |
| --- | --- | --- | --- | --- | --- | --- | --- | --- | --- | --- | --- | --- | --- |
| **WBH** |  |  |  |  |  |  |  |  |  |  |  |  |  |
| Drought | 2011 | 1.16±0.04 | 1.37±0.05 | 1.30±0.06 | 1.23±0.07 | 1.24±0.08 | 1.08±0.04 | 1.05±0.03 | 0.97±0.03 | 1.00±0.02 | 1.09±0.04 | 1.02±0.03 | 1.13±0.04 |
|  | 2014 | 1.11±0.05 | 1.06±0.04 | 1.11±0.04 | 0.90±0.03 | 0.97±0.03 | 0.93±0.03 | 1.06±0.03 | 0.97±0.03 | 1.03±0.03 | 1.06±0.03 | 0.95±0.02 | 1.00±0.03 |
|  | 2015 | 0.93±0.05 | 0.90±0.04 | 0.87±0.04 | 0.98±0.04 | 0.93±0.04 | 1.00±0.04 | 0.94±0.02 | 0.96±0.03 | 0.97±0.04 | 0.96±0.03 | 0.98±0.03 | 0.96±0.03 |
|  | mean Rt | 1.07±0.03 | 1.11±0.04 | 1.09±0.04 | 1.04±0.03 | 1.05±0.04 | 1.00±0.02 | 1.02±0.02 | 0.97±0.02 | 1.00±0.02 | 1.04±0.02 | 0.98±0.02 | 1.03±0.02 |
| Frost | 2010 | 0.61±0.02 | 0.59±0.02 | 0.59±0.02 | 0.73±0.03 | 0.66±0.03 | 0.75±0.02 | 0.77±0.02 | 0.79±0.02 | 0.75±0.02 | 0.69±0.03 | 0.76±0.03 | 0.65±0.02 |
| DBH [mm] | 2021 | 209±4.22 | 234±5.69 | 224±4.49 | 224±4.69 | 214±5.08 | 214±3.41 | 261±2.89 | 244±3.23 | 253±7.53 | 240±3.38 | 247±5.09 | 223±3.98 |
| **DDF** |  |  |  |  |  |  |  |  |  |  |  |  |  |
| Drought | 2003 | 0.78±0.02 | 0.71±0.03 | 0.79±0.03 | 0.78±0.02 | 0.77±0.04 | 0.81±0.03 | 0.73±0.02 | 0.77±0.02 | 0.75±0.02 | 0.76±0.02 | 0.75±0.03 | 0.79±0.02 |
|  | 2009 | 0.97±0.03 | 0.87±0.04 | 0.96±0.03 | 0.9±0.03 | 0.92±0.04 | 0.96±0.03 | 1.04±0.02 | 0.96±0.02 | 0.99±0.03 | 1.03±0.03 | 1.06±0.02 | 0.97±0.03 |
|  | 2018 | 0.85±0.03 | 0.90±0.05 | 0.91±0.05 | 1.03±0.05 | 1.02±0.06 | 1.01±0.05 | 0.83±0.03 | 0.87±0.02 | 0.90±0.03 | 0.85±0.04 | 0.84±0.03 | 0.88±0.03 |
|  | mean Rt | 0.87±0.02 | 0.83±0.03 | 0.89±0.02 | 0.90±0.02 | 0.91±0.03 | 0.93±0.03 | 0.87±0.02 | 0.87±0.02 | 0.88±0.02 | 0.88±0.02 | 0.89±0.02 | 0.88±0.02 |
| Frost | 2010 | 0.84±0.03 | 0.82±0.03 | 0.80±0.03 | 0.77±0.03 | 0.81±0.04 | 0.76±0.03 | 0.87±0.03 | 0.83±0.02 | 0.83±0.03 | 0.92±0.03 | 0.91±0.03 | 0.88±0.03 |
| DBH [mm] | 2021 | 237±4.22 | 189±3.21 | 201±4.85 | 221±4.51 | 226±7.65 | 203±6.02 | 269±5.57 | 263±4.02 | 276±5.74 | 280±4.61 | 266±5.37 | 269±5.33 |
| **WSD** |  |  |  |  |  |  |  |  |  |  |  |  |  |
| Drought | 2003 | 0.81±0.04 | 0.74±0.03 | 0.68±0.03 | 0.84±0.04 | 0.92±0.02 | 0.85±0.03 | 0.82±0.04 | 0.82±0.03 | 0.78±0.04 | 0.85±0.03 | 0.78±0.03 | 0.74±0.03 |
|  | 2016 | 1.13±0.05 | 1.27±0.05 | 1.32±0.06 | 1.06±0.05 | 1.02±0.05 | 0.94±0.04 | 1.01±0.03 | 0.93±0.04 | 1.05±0.04 | 1.02±0.05 | 1.02±0.04 | 1.07±0.03 |
|  | 2018 | 0.84±0.02 | 0.90±0.04 | 0.80±0.05 | 0.95±0.05 | 0.92±0.06 | 1.00±0.06 | 0.85±0.06 | 0.93±0.05 | 0.91±0.04 | 0.92±0.05 | 0.86±0.05 | 0.80±0.03 |
|  | mean Rt | 0.93±0.03 | 0.97±0.04 | 0.93±0.05 | 0.95±0.03 | 0.95±0.03 | 0.93±0.03 | 0.90±0.03 | 0.89±0.02 | 0.91±0.03 | 0.93±0.03 | 0.89±0.03 | 0.87±0.03 |
| Frost | 2010 | 0.55±0.03 | 0.59±0.02 | 0.56±0.02 | 0.61±0.02 | 0.62±0.02 | 0.66±0.02 | 0.60±0.02 | 0.66±0.03 | 0.61±0.02 | 0.61±0.03 | 0.64±0.02 | 0.63±0.03 |
| DBH [mm] | 2021 | 179±3.60 | 204±2.64 | 192±4.22 | 213±4.07 | 200±3.69 | 216±5.11 | 215±2.97 | 213±3.52 | 212±3.01 | 207±4.08 | 217±3.26 | 216±5.34 |

Supplementary Table 3: Results of ANOVA with resistance to late frost and drought as response and provenance, site and provenance x site interaction as fixed effects.

|  | Rt in frost year | | Mean Rt in drought years | |
| --- | --- | --- | --- | --- |
|  | p-value | Expl. Var [%] | p-value | Expl. Var [%] |
| Provenance | **< 0.001** | 6.1 | **0.044** | 1.1 |
| Site | **< 0.001** | 40.3 | **< 0.001** | 10.4 |
| Provenance x Site | **< 0.001** | 6.5 | 0.157 | 1.5 |
| Residuals |  | 47.1 |  | 87.1 |

# Supplementary Figures

**
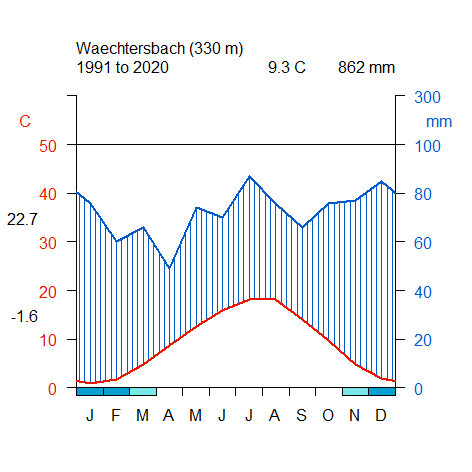

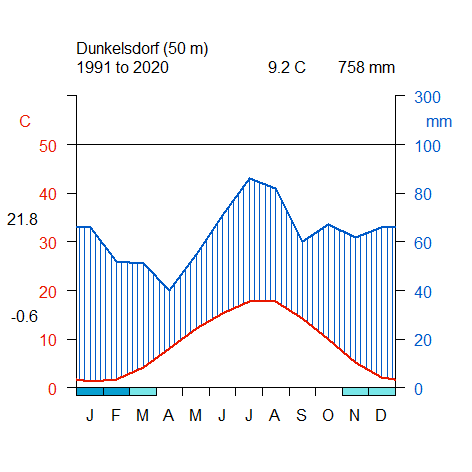

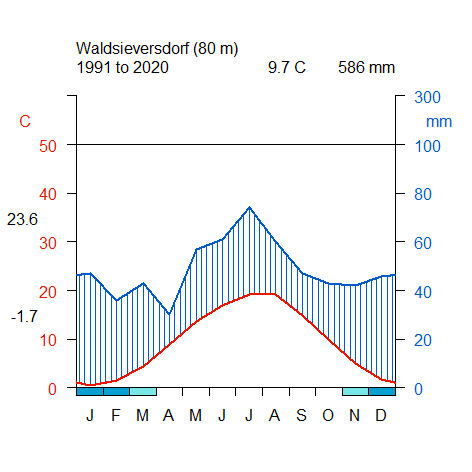
**

Supplementary Figure 1: Walter-Lieth diagrams for the three sites ordered by the precipitation gradient from wet (left) to dry (right).


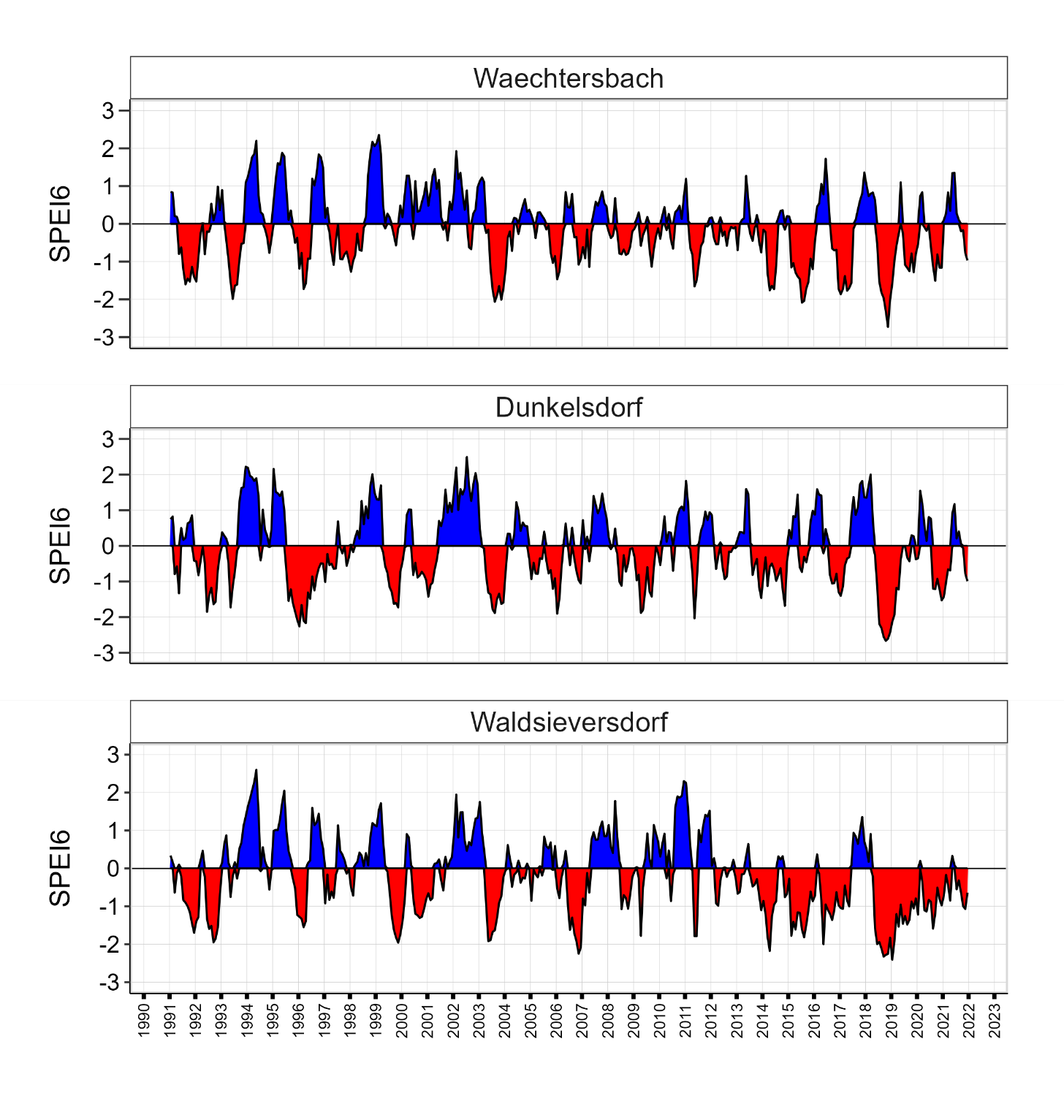


Supplementary Figure 2: SPEI6 during the time period from 1991 to 2021. Positive (blue) and negative (red) deviations are shown for each site.


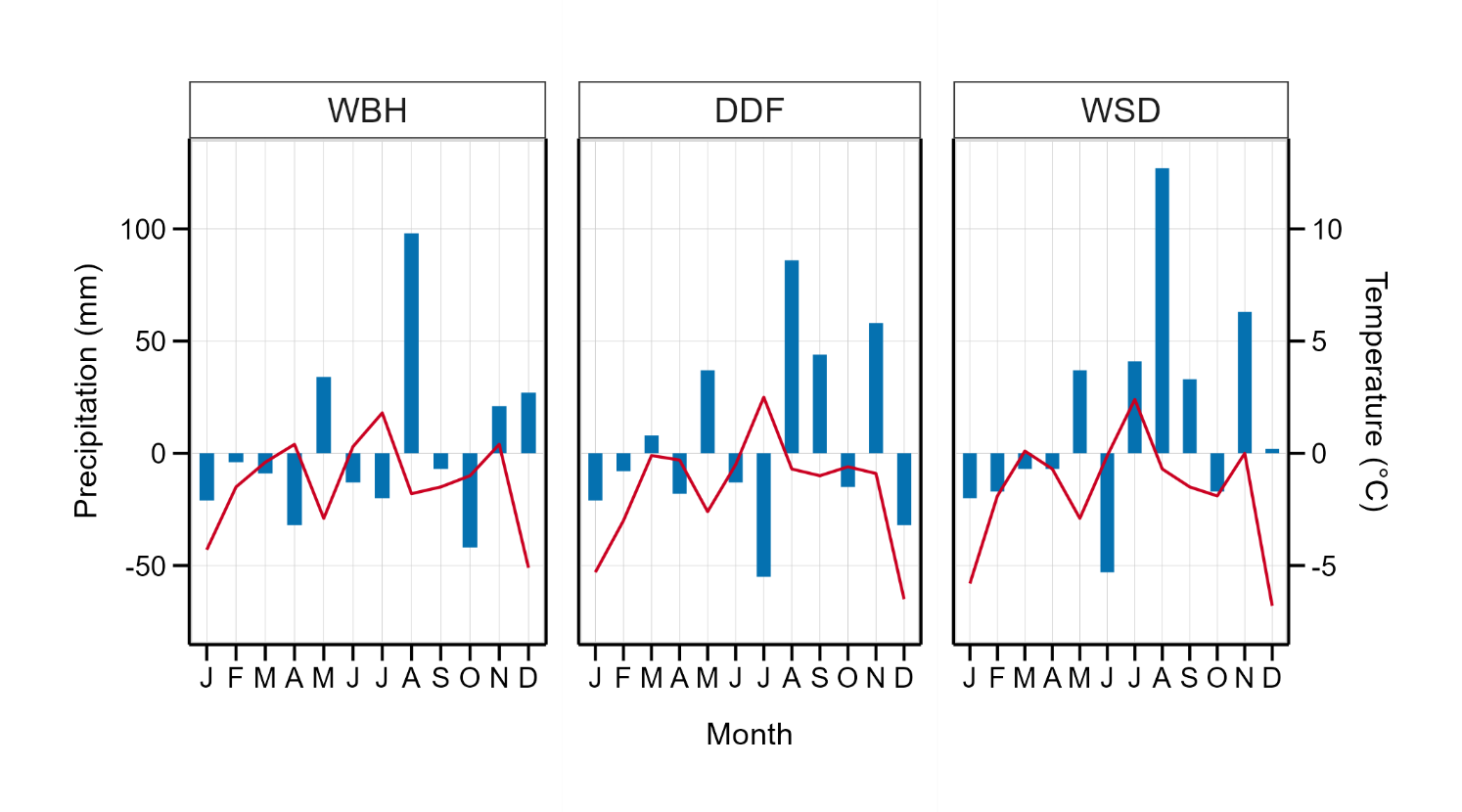


Supplementary Figure 3: Monthly climate anomalies in 2010. Blue bars represent deviations in precipitation and the red line in temperature.


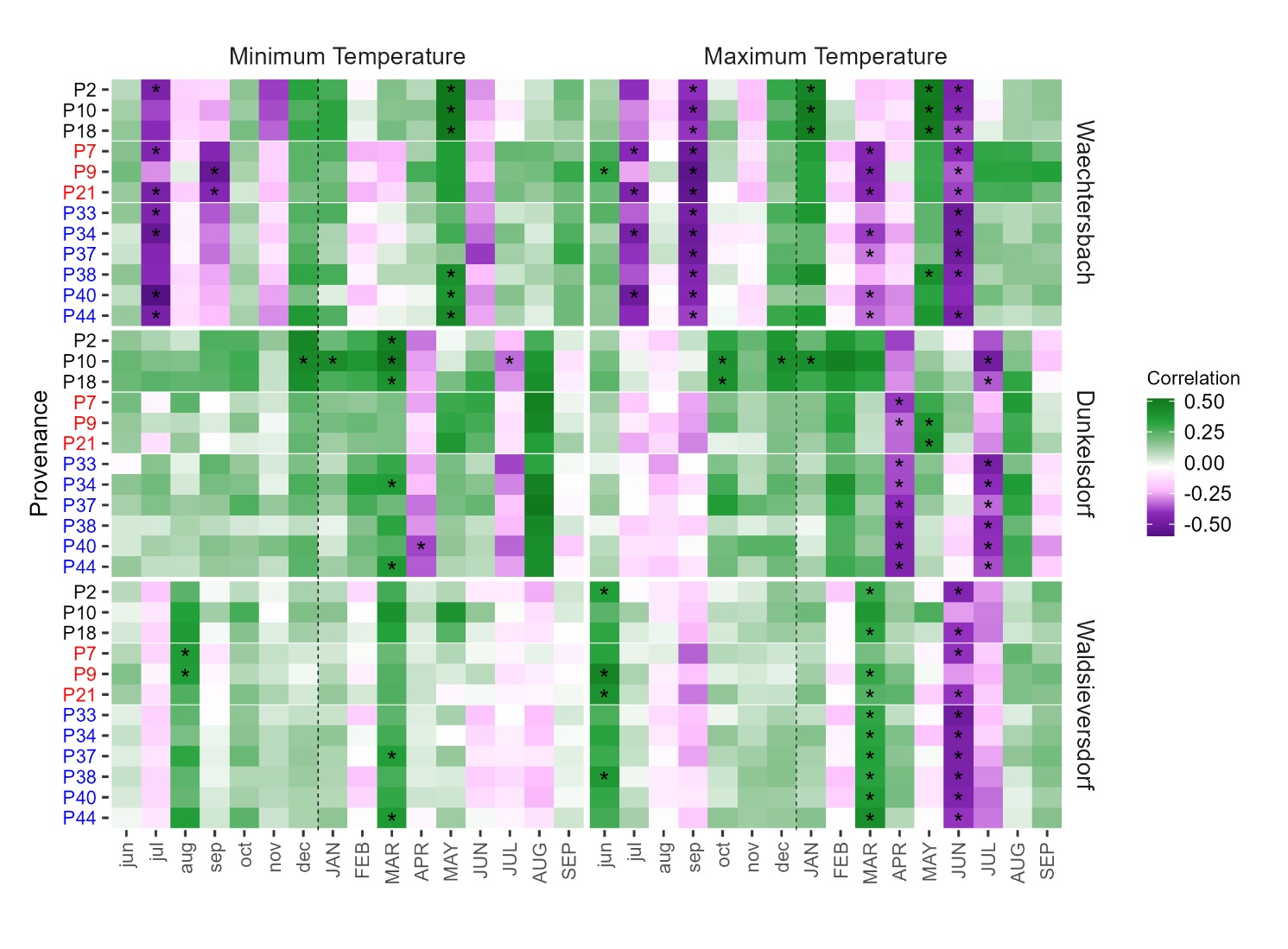


Supplementary Figure 4: Bootstrapped correlation coefficients for minimum and maximum temperature from previous June to current September on each site. Provenances are highlighted on the y-axis and grouped by county of origin (United States = black, Canada = red, Germany = blue). Correlation coefficients range from negative (violet) to positive (green) values and asterisks indicate significant correlations.
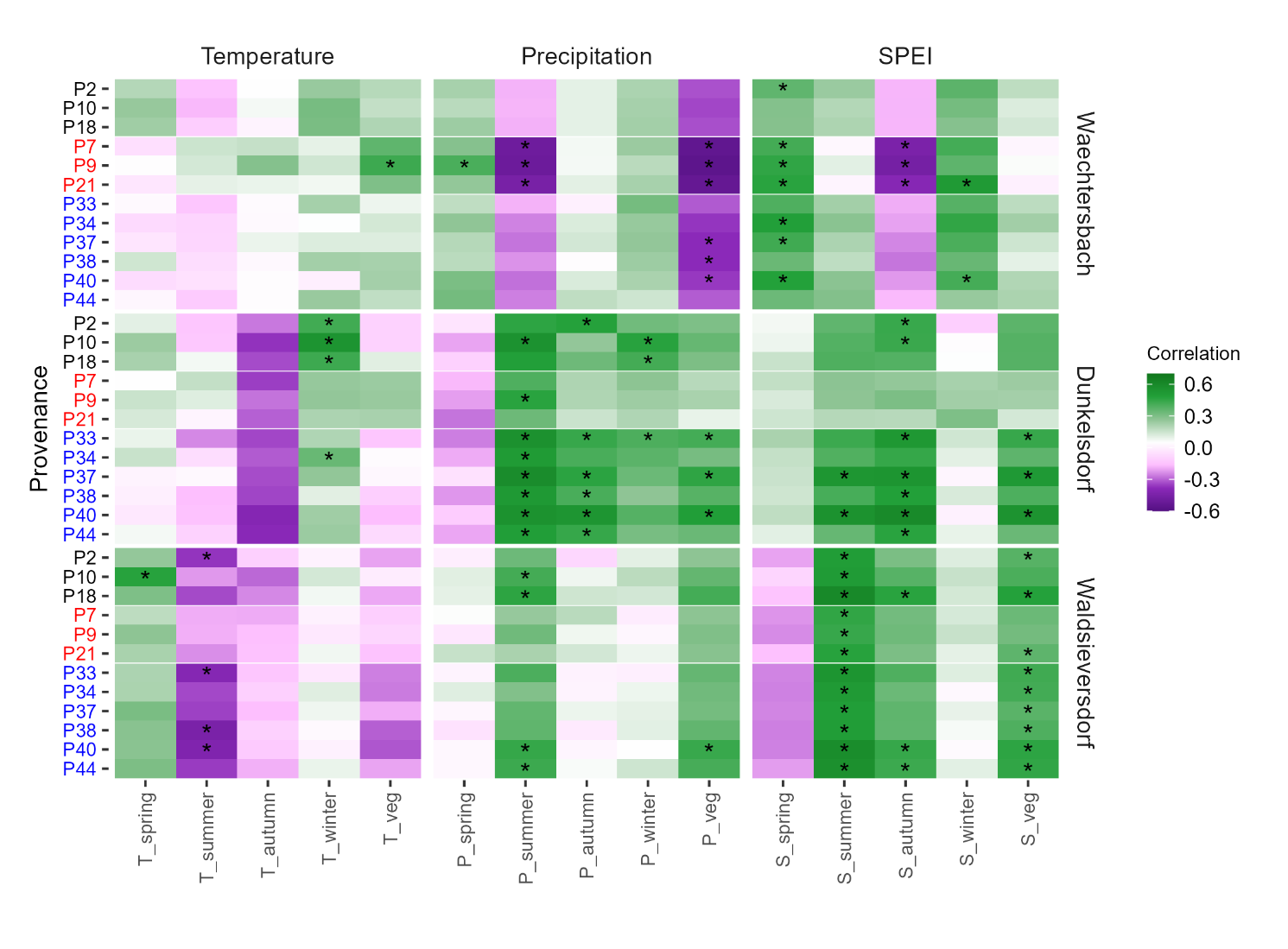


Supplementary Figure 5: Bootstrapped correlation coefficients for seasonal temperature, precipitation and SPEI as well as the vegetation period, respectively on each site. Provenances are highlighted on the y-axis and grouped by country of origin. Correlation coefficients range from negative (violet) to positive (green) values and asterisks indicate significant correlations.


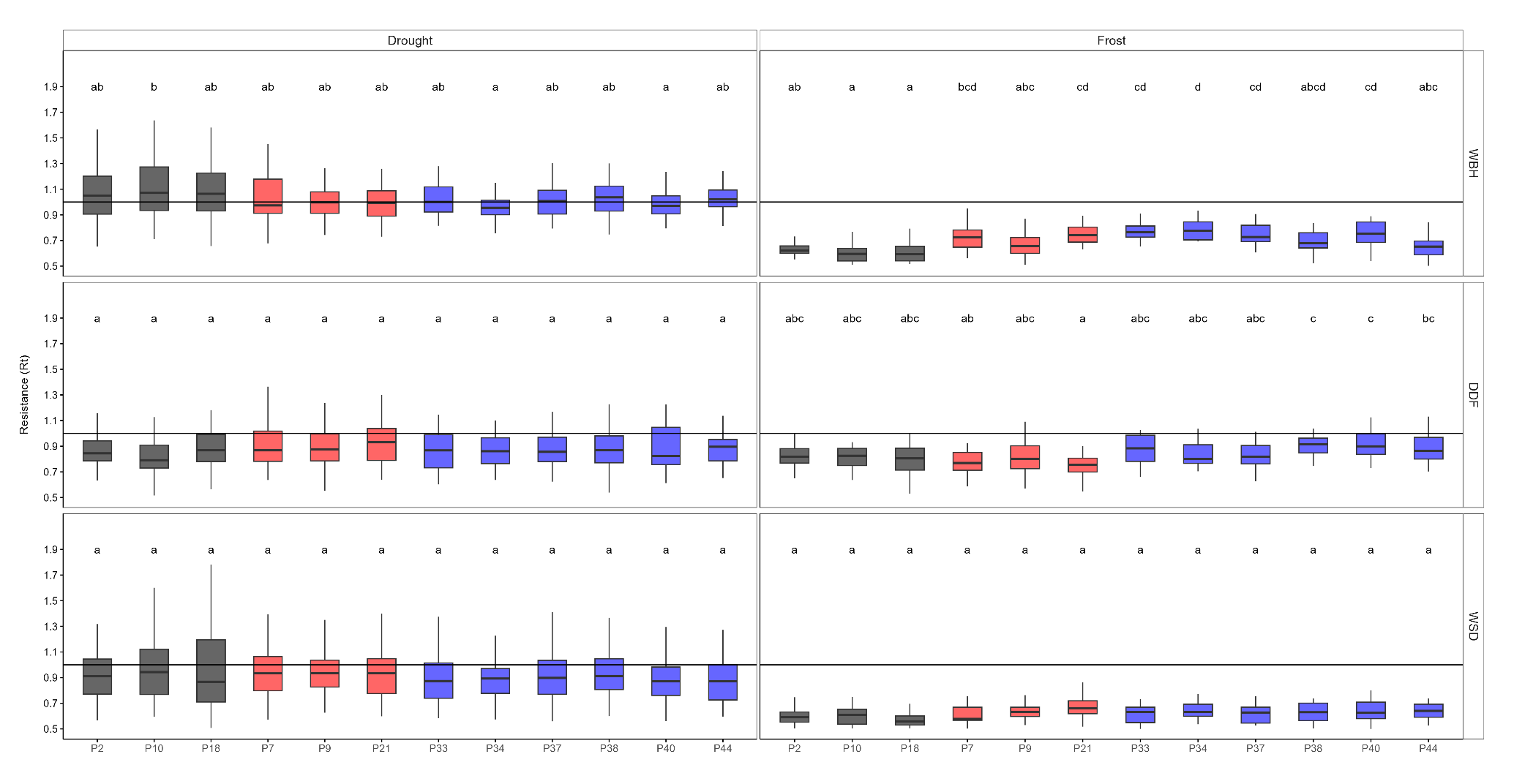


Supplementary Figure 6: Boxplots for resistance indices of individual trees per provenance, site and extreme event. Letters indicate differences between means according to Tukey's HSD post-hoc test. If the provenance means share the same letter, they cannot be shown to be different, but neither can they be shown to be the same. Sites are ordered according to the precipitation gradient from wet to dry.


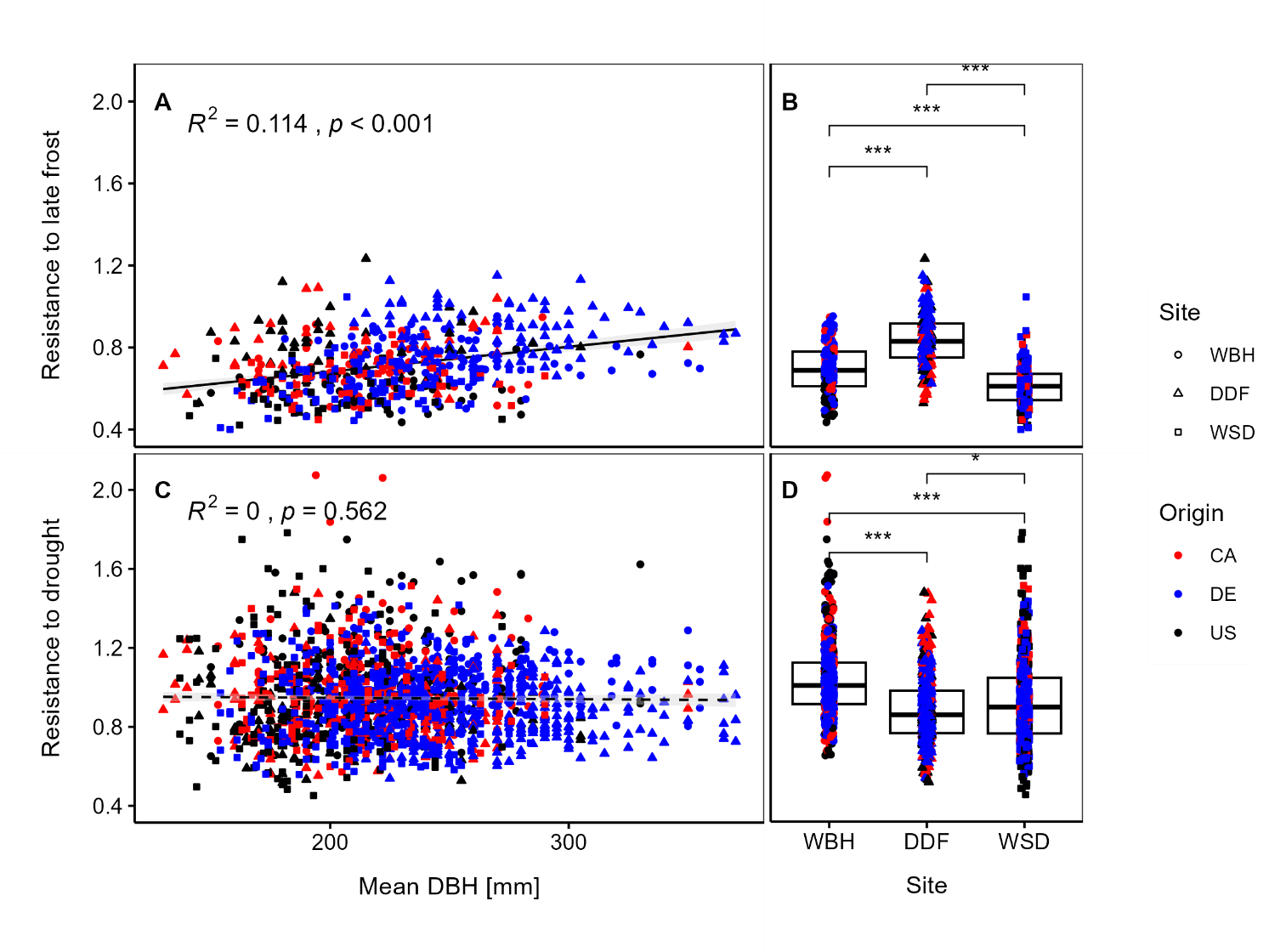


Supplementary Figure 7: Resistance (Rt) per individual tree to late frost (2010) plotted against the mean diameter at breast height (DBH) (A) and at each site (B). In the lower panels, resistance for site-specific drought years is plotted against the DBH (C) and at each site (D). Point shapes refer to the three sites and line types illustrate significant (solid) or non-significant (dashed) trends. Significant differences between the site means by Kruskal-Wallis test are indicated by * p < 0.05, ** p < 0.01, *** p < 0.001.
